# Advancing oncology drug therapies for sub-Saharan Africa

**DOI:** 10.1371/journal.pgph.0001653

**Published:** 2023-06-27

**Authors:** Kirthana Sharma, Tina Mayer, Sharon Li, Sadaf Qureshi, Faheem Farooq, Peter Vuylsteke, Tlotlo Ralefala, Richard Marlink

**Affiliations:** 1 Rutgers Global Health Institute, New Brunswick, New Jersey, United States of America; 2 Department of Medicine, Robert Wood Johnson Medical School, New Brunswick, New Jersey, United States of America; 3 Division of Medical Oncology, Rutgers Cancer Institute of New Jersey, New Brunswick, New Jersey, United States of America; 4 Rutgers Cancer Institute at University Hospital, New Jersey Medical School, Newark, New Jersey, United States of America; 5 Department of Internal Medicine, University of Botswana, Gaborone, Botswana; 6 Department of Oncology, Princess Marina Hospital, Gaborone, Botswana; PLOS: Public Library of Science, UNITED STATES

## Abstract

Cancer incidence is rising across sub-Saharan Africa (SSA), and is often characterized by late-stage presentation, early age of onset and poor survival. While a number of oncology drugs are now improving the length and quality of life for cancer patients in high-income countries, significant disparities in access to a range of oncology therapeutics exist for SSA. A number of challenges to drug access such as drug costs, lack of infrastructure and trained personnel must be urgently addressed to advance oncology therapies for SSA. We present a review of selected oncology drug therapies that are likely to benefit cancer patients with a focus on common malignancies in SSA. We collate available data from seminal clinical trials in high-income countries to highlight the potential for these therapeutics to improve cancer outcomes. In addition, we discuss the need to ensure access to drugs within the WHO Model List of Essential Medicines and highlight therapeutics that require consideration. Available and active oncology clinical trials in the region is tabulated, demonstrating the significant gaps in access to oncology drug trials across much of the region. We issue an urgent call to action to address drug access due to the predicted rise in cancer burden in the region in coming years.

## Introduction

Cancer incidence is rising across sub-Saharan Africa (SSA), and is often characterized by late-stage presentation, early age onset, aggressive disease and poor survival [1]. The distinct cancer epidemiology in SSA is driven by a high burden of infectious disease-related cancers, combined with rising lifestyle-related cancers due to factors such as increased tobacco use and excess body weight [2]. In addition, changes to female reproductive patterns and an ageing population are contributing to cancer risks [1]. Highly prevalent infectious diseases in SSA are causally associated with malignancies including cervical cancer and Kaposi sarcoma [3]. Cancers for which preventative measures are routinely offered in high income countries (HIC), such as breast, prostate and cervical cancer are advanced at presentation in SSA, because of a lack of organized screening and early detection programs [4]. In addition, evidence suggests that differences between tumour molecular subtypes prevalent in SSA impacts mortality in the absence of effective therapeutics [5]. This changing profile of risks and rising burden of cancer requires urgent and comprehensive approaches to expand access to oncology therapeutics in SSA.

Our understanding of the epidemiology of cancer in SSA is limited by a dearth in reliable cancer registries [6, 7], however, it is predicted that in SSA the cancer burden will double by 2050 [3]. Available data from cancer registries demonstrates increasing breast cancer incidence in 9 countries across the four SSA regions [3]. Registry data from Kampala, Uganda and Harare, Zimbabwe found a 4.5% and 4.9% average annual increase in breast cancer incidence, respectively. In addition, trends in 8 SSA countries over a 10-25-year period, found increasing incidence of cervical cancer, especially in Eastern Africa [8]. The cumulative risks and ASIR of prostate cancer have increased over time in the region [9]. For example, in Kampala, Uganda, an average annual percentage increase of 5.2% was found between 1991–2010 for prostate cancer [10]. Although few studies have discussed temporal trends in Kaposi Sarcoma (KS) in SSA, it is apparent that HIV-attributable KS is the highest in the region compared to other world regions [11]. Furthermore, the incidence of early-onset cancers is also rising globally. Risk factors implicated include multigenerational changes in diet, lifestyle, obesity, environment and the microbiome, and their interactions with genomic and/or genetic susceptibilities [12].

The rapid evolution of precision oncology therapies is increasingly transforming the length and quality of life for cancer patients in HICs. In SSA, however, in many settings, basic levels of cancer care, treatment and palliation are limited. Such inequities in treatment access contribute to the generally poor survival outcomes across most malignancies. In 2020, Africa’s contribution to global cancer mortality (7.2%) was greater than its contribution to global cancer incidence (5.7%), indicating higher case fatality rates compared with other regions [1]. Multiple barriers affect the ability of many cancer patients in SSA to access treatment and realize the benefits of advances in oncology drug therapies. These range from lack of infrastructure for molecular testing for driver mutations and biomarker expression, managing the unique set of adverse effects of newer drugs, such as immunotherapies, and shortages in healthcare workers adept in specialized cancer care. The cost of many new Food and Drug Administration (FDA) approved oncology drugs remains prohibitive, and there are extremely limited opportunities for access to these agents even in controlled clinical trials in the region. For patients, evidence suggests a lack of education and poor awareness of cancer signs and symptoms results in late-stage presentation. In Rwanda, lower education levels were significantly associated with lengthy delays to cancer diagnosis [13] and symptoms were attributed to benign co-morbid disease [13–15]. Seeking care from a traditional healer contributes to further delays and there is evidence that stigma and a lack of social support form additional barriers [16, 17]. Furthermore, geographic inequities in access to cancer facilities across SSA impacts cancer outcomes [18].

Despite these challenges, concerted efforts must be made to ensure inequities in access to cancer therapies are urgently addressed in SSA. Here, we review certain oncology drugs that are likely to have high-impact and durable benefit if expanded in SSA. We focus on cancers in SSA that have a high prevalence and high burden of disease including Kaposi sarcoma, prostate cancer, breast cancer, cervical cancer, and non-small cell lung cancer. In the context of advanced disease at presentation for most of these cancers in SSA, priorities are suggested to advance access to therapies in the region, with a focus on first-line treatments which have the greatest potential for therapeutic impact.

## Kaposi sarcoma

SSA carries a disproportionate burden of Kaposi sarcoma (KS) due to the endemic nature of the human herpes virus-8 and the human immunodeficiency virus (HIV) and acquired immunodeficiency syndrome (AIDS) epidemic. The incidence of KS in SSA has increased 20-fold since the onset of the HIV epidemic in the early 1980s [19]. KS is relatively rare globally, however, there were approximately 34,000 new cases and 15,000 deaths in 2020, largely in Southern and Eastern Africa. There are regional variations in SSA with highest age-standardised incidence rates (ASIR) found in Southern Africa approximating 9/100,000, compared with 6/100,000 in Eastern Africa, and 0.9/100,000 in Western Africa.^1^ Available data from the Africa Cancer Registry Network found a rapid increase in KS prior to antiretroviral therapy (ART), however, an overall average decline after ART between 2000 and 2010 and 2011–2016 [20]. While ART has reduced the incidence of AIDS associated KS, there remains ongoing significant morbidity and mortality from KS in the region [21].

Management for AIDS associated KS is driven by tumor location, extent of disease, CD4+ T cell count and overall clinical condition. Since most cases of KS that require intensive treatment are associated with HIV, it is imperative for all patients living with HIV to receive ART. Cutaneous lesions can be treated with ART, topical alitretinoin or imiquimod cream, radiation therapy, intralesional vinblastine chemotherapy, or local excision. However, in SSA, patients often present later in the disease course with advanced cutaneous, oral, visceral, or nodal disease. In HICs, chemotherapy with liposomal daunorubicin is recommended for advanced disease given its favorable toxicity profile.

Paclitaxel, however, has similar efficacy with 78% 2-year survival compared to 79% for liposomal daunorubicin (Table 1) [22]. A study of AIDS-associated Kaposi sarcoma in SSA and South America demonstrated the superiority of paclitaxel compared to etoposide, and bleomycin with vincristine. These findings were significant in that paclitaxel survival benefits may be comparable to liposomal daunorubicin, yet it is much more affordable and readily available in SSA [23]. As the first large clinical trial comparing chemotherapy for KS in SSA in over a decade, this study underscores the paucity of clinical trials for KS in SSA compared to the significant burden of disease in the region (Table 2). There is comparatively less interest in studying novel therapies for KS, however, one recently published trial of 17 patients in France demonstrated preliminary efficacy of pembrolizumab [24].

**Table 1:** Selected review of high impact clinical trials* related to recommended current standard of care for common malignancies.

Tumor Type	Drug	Overall Survival (OS)	Progression-free Survival (PFS)	Dates of patient enrollment	Study Population	n	Trial (Reference)
Kaposi Sarcoma	Paclitaxel vs. pegylated liposomal doxorubicin	At 2 years, OS 79% vs. 78%	17.5 vs 12.2 mo	1998–2002	HIV associated KS	73	Cianfrocca et al. Cancer. 2010
	Paclitaxel vs. intravenous bleomycin + vincristine vs. oral etoposide		At 48 weeks, 64% vs. 50% vs. 20%	2013–2018	Advanced AIDS associated KS	334	Krown, et al. Lancet 2020
Prostate	Abiraterone + Prednisone vs. Placebo	53.3 months vs. 36.5 months with placebo; HR 0.66		2013–2014	Newly diagnosed metastatic castrate sensitive	1199	LATITUDE
	Docetaxel + ADT vs. ADT. alone	58 versus 47 months With ADT alone; HR 0.72	33 vs. 14.8 mo; HR 0.47	2006–2012	Newly diagnosed metastatic castrate sensitive	790	CHAARTED
	Abiraterone + Prednisone vs. ADT alone	83% versus 76% with ADT alone at 3 years; HR 0.63		2011–2014	Newly diagnosed metastatic castrate sensitive	1917	STAMPEDE
	Enzalutamide + ADT vs. ADT alone	80% vs 72% at 3 years	Clinical Progression—EFS at 3 years, 68% and 41%, HR 0.40; PSA progression–EFS at 3 yrs—67% vs. 37; HR -0.39	2014–2017	Newly diagnosed metastatic castrate sensitive	1125	ENZAMET
	Apalutamide + ADT vs. ADT alone (prior docetaxel allowed but not concurrent)	82% vs 74% at 2 years, favoring apa HR 0.67	rPFS at 24 months 68% vs 48% favoring apa HR 0.48	2015–2017	Newly diagnosed metastatic castrate sensitive	1052	TITAN
Breast	Pertuzumab + Trastuzumab/Docetaxel vs. Trastuzumab/Docetaxel alone	41.8% vs. 54.4%; HR 0.6	18.7 vs. 12.4 mo; HR 0.68	2008–2010	HER2+ metastatic, unresectable, or locally recurrent breast cancer	808	CLEOPATRA
	Trastuzumab for 1 year, Trastuzumab for 2 years, or observation only	Survival rates at 12 years were 79% in the 1-year group, 80% in the 2-year group, and 73% in the observation group	Rates of 10-year disease-free survival were 69% in both trastuzumab groups and 63% in the observation group.	2001–2005	HER2-positive breast cancer after standard adjuvant chemotherapy	5081	HERA
	Trastuzumab deruxtecan vs. Trastuzumab emtansine	At 12 months, the percentage of patients who were alive without disease progression, as assessed by blinded independent central review, was 75.8% (95% CI, 69.8 to 80.7) with trastuzumab deruxtecan as compared with 34.1% (95% CI, 27.7 to 40.5) with trastuzumab emtansine	Median PFS was not reached (95% CI, 18.5 to could not be estimated) in the trastuzumab deruxtecan group and was 6.8 months (95% CI, 5.6 to 8.2) in the trastuzumab emtansine group.	2018–2020	HER2-positive metastatic breast cancer after progression on taxane and trastuzumab (second line)	524	DESTINY-Breast03
	Anastrazole/Letrozole + Abemacicib vs. placebo	46.7 vs. 37.3 mo, HR 0.757	6.4 vs 9.3 mo., HR, 0.553	2014–2015	HR+/HER2-negative advanced breast cancer	669	MONARCH-2
	Fulvestrant + Ribociclib vs, placebo	57.8% vs. 45.9% at 42 mo, HR 0.72	33.6 vs. 19.2 mo, HR 0.55	2015–2016	HR+/HER2-negative advanced breast cancer	726	MONALEESA-3
	Fulvestrant + Palbociclib vs. placebo	39.7 mo. vs. 29.7 mo., HR 0.72	9.5 vs. 4.6 mo, HR 0.46	2013–2014	HR+/HER2-negative advanced breast cancer	521	PALOMA-3
	Atezolizumab plus nab-Paclitaxel vs. placebo plus nab-Paclitaxel	21.3 mo vs. 17.6 mo; HR 0.84	7.2 mo vs. 5.5 mo; HR 0.8	2015–2017	Untreated locally advanced or metastatic TNBC	902	IMpassion130
Cervical	Cisplatin + Paclitaxel vs. Cisplatin + Topotecan	15 vs. 12.5 mo, HR 1.2	7.6 v. 5.7 mo, HR 1.39	2009–2012	Metastatic or recurrent	452	GOG-240
	Chemotherapy (Paclitaxel + Cisplatin or Carboplatin +/- Bevacizumab) + Pembrolizumab vs. Placebo	53% vs. 42% at 24 mo, HR 0.64	10.4 vs. 8.2 mo, HR 0.62)	2018–2020	PD-L1 positive metastatic or unresectable, not previously treated in the recurrent or metastatic setting	548	KEYNOTE-826
Lung	Pembrolizumab + chemotherapy	61.7% vs. 52.2% at 12 mo; HR 0.55	34.1% vs. 17.3% at 12 mo; HR <1	2016–2017	metastatic non-squamous NSCLC without sensitizing EGFR or ALK mutations who had received no previous treatment for metastatic disease	616	KEYNOTE-189
	Osimertinib vs. comparator TKIs	38.6 mo vs. 31.8 mo; HR 0.8	18.9 mo vs. 10.2 mo; HR 0.46	2014–2016	previously untreated advanced NSCLC with an EGFR mutation (exon 19 deletion or L858R allele	556	FLAURA

*Most studies have been performed in HICs

Legend: OS–overall survival; PFS–progression free survival; HR–hazard ratio; ADT–androgen deprivation therapy; KS–Kaposi Sarcoma; NSCLC–non small cell lung cancer; mo—months

**Table 2:** Overview of oncology drugs in the WHO model list of essential medicines for common malignancies in sub-Saharan Africa.

	WHO MLEM oncology therapies	Biosimilars/ generic availability	Patent expiry date	WHO recommended essential diagnostic tests	Number of active pharmacologic intervention trials by cancer in SSA (as of 10/31/21)
Kaposi Sarcoma	Bleomycin Doxorubicin Paclitaxal Vincristine Vinblastine (topical)	Generics available for all		N/A	3 (3 in Uganda, 1 each in South Africa, Malawi, Kenya)
Prostate	Abiraterone Bicalutamide Docetaxel Leuprorelin Prednisolone	Generics available for all	Abiraterone—2027	Prostate specific antigen (PSA)	6 (6 in South Africa)
Breast	Anastrazole Carboplatin Cyclophosphamide Docetaxel Doxorubicine Fluorouracil Leuprorelin Methotrexate Paclitaxel Tamoxifen Trastuzumab*	Generics available for all *Trastuzumab biosimilar available		Estrogen and progesterone receptors via IHC; ERBB2/HER2 expression via IHC	15
Cervical	Carboplatin Cisplatin paclitaxel	Generics available for all		Pap smear	2 (1 in South Africa, 1 in Ghana)
Lung	Carboplatin Cisplatin Erlotinib Etoposide Gemcitabine Paclitaxel Vinorelbine	Generics available for all	Erlotinib -2020 (expired)	N/A	14 (14 in South Africa)

Legend: N/A- not applicable; IHC- immunohistochemisty

## Prostate cancer

Prostate cancer is the leading cause of cancer death among men in 48 countries, including many in SSA [19]. There is, however, substantial regional variation in the incidence of prostate cancer across SSA, with the highest ASIR in the region found in Southern Africa approximating 66/100,000 in 2020, compared with Western and Eastern Africa where ASIR approximates 30/100,000 [1]. Mortality rates are high in SSA, with age-standardized mortality rates (ASMR) in 2020 approximating 22/100,000, 20/100.000 and 16/100,000 in Southern, Western and Eastern Africa respectively. While data are very limited, a median overall survival of only 11 months was reported for metastatic castrate resistant prostate cancer (mCRPC) in a series from Nigeria [25]. Treatment with surgical castration is widespread in SSA, with limited use of luteinizing hormone releasing hormone (LHRH) analogues, oral anti-androgens (bicalutamide and flutamide), or docetaxel [26, 27]. For metastatic prostate cancer, androgen deprivation therapy can provide symptom relief and improve survival. The disease invariably becomes resistant to hormone therapy however, leading to development of mCRPC.

Newer generation oral hormone therapy may have an expanded role in SSA, in both the castrate-sensitive and castrate-resistant settings. Abiraterone is a well-tolerated drug which blocks synthesis of androgens and is given in combination with low dose prednisone. Based on data showing significant survival benefit, abiraterone with prednisone was initially approved by the US FDA in 2011 for patients with mCRPC who had received prior chemotherapy and then expanded to chemotherapy-naive patients. Due to significant benefits in the castrate-sensitive group, indications were expanded in 2018 for patients with newly diagnosed castrate-sensitive metastatic prostate cancer. Overall, the therapy was well tolerated with notable side effects being hypertension and hypokalemia [28]. Updated data from the STAMPEDE study demonstrated a median survival of 6.6 years with addition of abiraterone with prednisone to standard androgen deprivation therapy (ADT), compared with 3.8 years with ADT alone [29]. Survival benefits have also been demonstrated with addition of either enzalutamide or apalutamide to ADT in patients with newly diagnosed mCRPC (Table 1). The addition of chemotherapy with docetaxel also has a demonstrated survival benefit [30]. The emerging evidence clearly demonstrates that intensifying therapy for men with metastatic prostate cancer earlier in disease course, even in the castrate-sensitive setting, has clear survival advantage.

PARP inhibitors, such as olaparib, have a role in patients with mCRPC with certain molecular profiles, particularly BRCA 2 [31]. Molecular profiling capabilities are, however, required, and the extent of BRCA 2 mutations in SSA populations remains poorly characterized at present. Therapeutic options including chemotherapy, immunotherapy and radiopharmaceuticals also clearly have an established role for management of mCRPC, however rapidly expanding availability of newer generation oral hormone therapy is likely to have important impacts. This is reflected by the inclusion of abiraterone with prednisone in the World Health Organization (WHO) Model List of Essential Medicines (MLEM) (Table 2). Though enzalutamide and apalutamide also have proven benefit in the castrate-sensitive metastatic prostate cancer setting, abiraterone may be preferred for several reasons. As the first of the newer generation hormone therapies to become available, abiraterone will likely have multiple generic options available first, with hopes of reduced pricing in the future. Cost savings could also be achieved by alternative dosing with food [32].

## Breast cancer

The incidence rates of breast cancer in SSA are low compared to HICs, however, these rates are rapidly rising [6]. In Southern Africa ASIR approximated 50/100,000 in 2020 with mortality rates among the world’s highest due to late-stage presentation and lack of screening programs [1]. In addition, evidence suggests a higher percentage of hormone receptor negative or triple negative disease in the region, both of which are associated with a poorer prognosis [33].

Treatment for breast cancer greatly differs based on hormonal status and human epidermal growth factor (HER2) expression status. Accurate profiling, however, is dependent on methods used for tissue collection, fixation, and immunohistochemical staining, which are not standardized in SSA. Hormonal therapy, including drugs such as tamoxifen and aromatase inhibitors (anastrozole, letrozole, or exemestane), is indicated for hormone-receptor positive tumors. While access to aromatase inhibitors remains limited, tamoxifen is inexpensive and even free of charge in some countries, making it the most readily available drug for breast cancer in SSA [33–35]. Consequentially, patients whose hormone receptor status is unknown may empirically receive tamoxifen. While routine testing for hormone receptor status prior to treatment initiation is uncommon, in a recent meta-analysis of five East African countries, 55% of women with breast cancer were estrogen receptor (ER) positive compared to 72% in the United States [36]. However, only those who are hormone receptor positive are expected to benefit from treatment with tamoxifen, hence patients are often exposed to the potential harms of tamoxifen with no therapeutic advantage [37, 38].

The predominant chemotherapy regimens for patients with HER2 negative disease are anthracycline based regimens such as doxorubicin and cyclophosphamide followed or preceded by a taxane (paclitaxel or docetaxel), or anthracycline free regimens such as docetaxel and cyclophosphamide. The WHO MLEM includes agents such as carboplatin, cyclophosphamide, doxorubicin, and paclitaxel, but availability and access to these drugs appears limited. For example, in a population-based registry study of 834 patients in 11 countries in SSA, only 33.6% received chemotherapy, of which 52% received an anthracycline based regimen and 32% received an anthracycline with a taxane [39].

The advent of HER2 targeted drugs have transformed the management and prognosis of HER2 positive breast cancer in HICs. The HERA trial established the role of trastuzumab after adjuvant chemotherapy in HER2 positive breast cancer patients by demonstrating significantly improved disease-free survival (DFS) and overall survival (OS) [40, 41]. Unfortunately, trastuzumab has historically been cost-prohibitive [42]. While some studies estimate that 50% of facilities in SSA have trastuzumab available, less than 5% of patients were able to afford it [33]. The advent of biosimilars since 2019, however, are now facilitating price reductions to $600–800 dollars per vial in SSA countries (Table 2) [43].

There is a high incidence of triple negative breast cancer (TNBC) in SSA, and this breast cancer pathologic subtype is negatively associated with survival [44, 45]. Two immunotherapy drugs, atezolizumab and pembrolizumab, are approved for use in the US in combination with chemotherapy for patients with metastatic TNBC whose tumors have high expression of PD-L1, based on the results of the IMpassion130 and KEYNOTE-355 trials [46, 47]. Unfortunately, predictive biomarker testing that is required to identify patients with PD-L1 positive tumors is not widely available in SSA. In addition, immunotherapy is associated with rare but serious adverse events that require routine monitoring and specialized management. Therefore, there is a need to develop further training and infrastructure to support the use of these effective drugs.

Other targeted drugs are integral parts of the treatment landscape for metastatic breast cancer, such as targeting PI3Kinase, mammalian target of rapamycin, and cyclin dependent kinases (CDKs). Of these drugs, the CDK 4/6 inhibitors have shown the greatest benefit and the fewest side effects. The addition of CDK4/6 inhibitors to hormonal therapy with aromatase inhibitors or fulvestrant showed a significant and clinically meaningful survival benefit in first- and second-line metastatic settings (see Table 1) [48–50]. Given the limited toxicity profile of CDK4/6 inhibitors compared to chemotherapy and their oral administration, they have the potential to improve care more widely in SSA (see Table 2). The challenge for implementation is the elevated cost of CDK4/6 inhibitors, as well as the limited availability of tumor profiling.

## Cervical cancer

In SSA, cervical cancer (CC) is the leading cause of female cancer related death and the second most common cancer among women [4, 8]. Globally, the highest incidence and mortality rates are in SSA with ASIR of 40/100,000 in Eastern Africa and 37/100,000 in Southern Africa in 2020 [1]. This higher incidence in SSA parallels HIV-infection rates–as an AIDS-defining illness HIV-positive women have a 6-fold higher risk of CC due to higher infection and persistence rates of cervical oncogenic Human Papilloma Virus (HPV) genotypes. Primary prevention through adolescent HPV vaccination programs have been launched in SSA, however, will take several decades to impact CC incidence [51]. In addition, expanding coverage of accessible, affordable, and high-performance cervical screening programs has been challenging due to financial, logistic, and sociocultural barriers [52]. Due to the preventable nature of CC, the WHO has launched a strategy for the global elimination of cervical cancer focusing on rapid scale-up of vaccination, screening and treatment of pre-cancers and CC.

The standard of care for management of patients with locally advanced CC disease is chemoradiation. The preferred chemotherapy in conjunction with radiation is cisplatin [53]. A study which included twenty-nine centers from twelve SSA countries found that appropriate chemotherapeutic agents were available in 86% of centers. However, one-third of centers reported having to delay treatments or substitute with alternate chemotherapeutic agents due to inconsistent supply. In addition, almost half of the centers utilized neoadjuvant chemotherapy due to delays in availability of surgery and radiotherapy [54].

Radiotherapy is integral to the management of advanced CC but there is limited access in SSA due to the need for trained personnel for equipment maintenance and optimal implementation [55]. In addition, maintenance and repair of linear accelerators and lack of access to a stable and reliable power supply may result in downtime of equipment, ultimately leading to treatment delays for patients [56]. In an observational study of 632 patients from eight SSA countries, only 13% of patients with known stage I–III CC received primary external beam radiation therapy and brachytherapy [57].

In addition to concurrent chemoradiotherapy for localized disease, chemoimmunotherapy (with pembrolizumab in combination with a platinum doublet) was FDA approved in October 2021 for persistent, recurrent, or metastatic cervical cancer patients whose tumors are PD-L1 positive [58]. Given many women progress while waiting for radiotherapy, improving access to testing for PD-L1 status and the capacity of providers with expertise in managing immunotherapy, should be considered to expand the feasibility of this option in SSA.

## Lung cancer

The burden of lung cancer in SSA is expected to increase in parallel to the tobacco epidemic, with greater affordability and marketing of tobacco products in the region [4]. In SSA, lung cancer rates are highest in Southern Africa with ASIR of 27/100,000 in 2020 compared with Eastern Africa with ASIR of 4/100,000 [1]. In HICs, molecular targeted therapies for lung cancer have achieved substantial survival benefits. Current standards for management of lung cancer involve first identifying histologic subtype via immunohistochemistry (IHC) to differentiate between small cell (SCLC), non-small cell (NSCLC), or squamous cell lung cancer (SLC). In most HICs, testing NSCLC for mutations in EGFR, BRAF, KRAS, and ALK and ROS1 gene rearrangements via liquid biopsy, next-generation sequencing (NGS), and/or fluorescent in-situ hybridization is common [59, 60]. At a minimum, mutations in EGFR, ALK status, and PDL1 status via IHC should be known before starting first-line systemic therapy because regimens for patients with these mutations can differ drastically [59].

Traditionally, recommended treatment for patients with metastatic NSCLC has been chemotherapy with a platinum-based combination regimen. However, median survival for patients receiving chemotherapy alone approximates to 10 months, with 1-year survival of only 30–40%, and 5-year overall survival of approximately 6% [61–66]. Survival rates are higher for stage IV NSCLC patients who are eligible for either targeted therapy or immunotherapy regimens, which increased overall survival from 10 to 24 months in those receiving EGFR- tyrosine kinase inhibitor (TKI) therapy versus those who did not [67–72]. Afatinib, erlotinib and gefitinib are therefore included in the WHO MLEM. However, patients often develop mutations which result in resistance to these agents. Osimertinib, which inhibits both EGFR sensitizing mutations and T790M, a common resistance mutation in EGFR mutated NSCLC, was approved in 2020 in the United States, however, is not listed in the WHO MLEM at present. Osimertinib, has achieved median overall survival exceeding 45 months, far greater than other TKIs. [73]. The notable impact of osimertinib for NSCLC suggests that it should be the TKI of choice and considered in the WHO MLEM.

In the population of stage IV NSCLC patients without a driver mutation, immunotherapy with pembrolizumab is recommended either as a single agent in patients with high PDL1 expression, or in combination with chemotherapy in those with low PDL1 expression. In patients with PDL1 expression in at least 50% of tumor cells, overall survival is doubled in patients who receive pembrolizumab monotherapy compared with those who receive chemotherapy (30 vs 14.2 months) [74]. Long-term data from trials have shown 5-year survival for patients upwards of 23% for those who received first-line pembrolizumab monotherapy [66, 68]. Overall survival was also improved by chemotherapy regimens combined with pembrolizumab regardless of PD-L1 expression levels [75].

The number of agents that are approved and under investigation for NSCLC are increasing worldwide, however, access remains extremely limited in most settings in SSA (Table 2) [76]. One review of lung cancer management in South Africa noted that for private sector patients with EGFR mutations, only erlotinib is available for first line use, osimertinib is available in the second line setting and crizotinib is the only drug available for ALK positive patients [76].

For management of NSCLC to be optimal in SSA, identifying mutations in EGFR and PDL1 expression are essential to direct care. In addition, EGFR inhibitors, particularly osimertinib, and immunotherapy, particularly pembrolizumab, are two targeted therapies that have the potential to improve outcomes of NSCLC patients in SSA if given as first-line.

## Tissue agnostic therapeutics for multiple solid tumors

Both hereditary and sporadic cancers arising at several anatomical sites including colon, rectum, endometrium, ovary and gastric adenocarcinoma may have mismatch repair deficiency (dMMR) or microsatellite instability-high (MSI-H), which have treatment implications [77]. In an analysis of MSI-H in 39 tumor types across 11,139 samples, MSI-H was found in 31% of endometrial cancers, 19% of stomach and 19.7% of colon adenocarcinomas [78]. While far more research is needed, available data suggests even higher rates of MSI-H were found in solid tumors from SSA. For example, in a sample of 90 colorectal cancer biospecimens in Ghana and 83 specimens in Nigeria, MSI-H was found in 41% and 43% of samples respectively [79, 80]. Another study of 83 cases from KwaZulu Natal in South Africa, found MSI-H in 31.3% of colorectal cancer patients below age 35 years, and 12% in cases over 50 years [81].

The US FDA granted accelerated approval of pembrolizumab in May 2017 for patients with advanced previously treated solid tumors found to be MSI-H which has heralded the potential for histology-agnostic management regardless of site of origin for solid tumors [82]. Pembrolizumab approval was based on safety and efficacy results in 149 patients with MSI-H solid tumors that had progressed after standard treatment, who were enrolled in one of five multi-center, single-arm clinical trials (KEYNOTE-016, -164, -012, -028, and -158) [83]. The overall response rate was 39.6%, and importantly, responses lasted six months or more for 78% percent of responders, with 7.4% achieving complete responses [84].

Therapeutics that are tissue -agnostic raise the prospect of enabling the use of a select range of drugs across a wide range of solid tumors, with potential advantages in terms of costs, training and infrastructure needs in resource limited settings in SSA, especially if research suggests higher rates of MSI-H among SSA populations. A small number of trials of pembrolizumab which are histology-agnostic are, therefore, being conducted in South Africa evaluating gastro-intestinal and gynecological cancers.

## Discussion

In SSA, the choice of public sector pharmaceuticals is often guided by the WHO MLEM, which are selected based on three broad criteria including disease prevalence and public health relevance, evidence of clinical efficacy and safety, and comparative cost-effectiveness. In addition, resource stratified guidelines are published by the National Comprehensive Cancer Network, however, evidence regarding the translation of guidelines into real-world practice is lacking. A survey of oncologists across low-, middle-, and high-income nations demonstrated concordance with the WHO MLEM but found significant barriers to the availability of therapies included in the WHO MLEM [85]. While even basic levels of palliative care or consistent supply of cytotoxic agents are still not widely available in many SSA settings, the inclusion of immunotherapies in the 2019 WHO MLEM signals an increasing recognition of the potential advantages of precision therapeutics in LMICs. While the 2019 MLEM only listed immunotherapy for metastatic melanoma due to high prices and concerns regarding effective administration in settings without appropriate infrastructure, one could argue that if capacity is built for treatment of melanoma, wider indications to NSCLC and other solid tumors will be feasible, widening the population for which quality of life and survival benefits may be realized.

The availability of molecular profiling of cancers if of paramount importance, particularly for certain cancers such as lung cancer for which targeted therapies have had a major impact on prognosis.

The presence of BRCA1/2 mutations in breast and prostate cancer, as well as BRAF mutations in melanoma, are other examples where molecular profiling can impact cancer therapeutics. Unfortunately, data on genomic profiles of cancer in patients in SSA is extremely limited [86]. Of worldwide cancer publications addressing genomics, only 0.016% evaluated people from SSA. Authorship by researchers based in SSA was limited even in manuscripts evaluating African populations, which was noted as a barrier to advancing the knowledge base in this field [87]. Rotimi et al performed a comprehensive literature review evaluating the published literature on cancer genomics in African populations between 1990–2019. Overall, there was a paucity of data, with the majority of SSA populations remaining largely unstudied. While breast cancer was studied most extensively, studies were predominantly limited to populations in North Africa which may not be representative of SSA. BRCA1 and BRCA2 mutations were noted in high frequency in Nigerian women, and some studies included South African and Senegalese populations. Data regarding genomic alterations in populations of prostate cancer patients in SSA was minimal, including data on frequency of BRCA1, BRCA2 or other homologous recombination repair genes [88]. For lung cancer, most notably EGFR, ALK and PD-L1 expression has had a significant impact on management, however, there is very limited data available of molecular profiles of lung cancer patients in SSA. Available data on EGFR expression in African populations is limited to North African populations [89].

We recognize that costs and cost-effectiveness concerns are important factors in realistically increasing availability of a broad range of oncology drug therapies in SSA. The moral need, however, to advance therapeutics and reduce the significantly high case-fatality rates from cancer in SSA remains an urgent global imperative. As such, the need for high levels of investment in cancer care has been acknowledged by all member states of the World Health Assembly in 2017, with the scale up national cancer prevention and control aligned to achieving the 2030 Agenda for Sustainable Development. To this end, several global organizations are challenging cost pressures such as the WHO medicines patent program, pooled procurement approaches, voluntary licensing and there are moves by the World Economic Forum to create enabling governance, regulatory and ethical environments to broaden the reach of precision therapies and diagnostics world-wide [90]. There is a need to harness the global drive to develop newer therapeutics that can transform cancer care, and global interest in including more diverse patient populations in clinical trials. Historically, a number of barriers including laboratory infrastructure, trained personnel and slow regulatory approval processes have delayed successful implementation of oncology clinical trials in SSA [91]. Future efforts, however, must expand clinical trial capacity to ensure inclusion of SSA populations in new and pre-clinical studies to improve access to the pipeline of new therapeutics, while forging avenues to expand access to existing approved therapeutics in SSA. Furthermore, in coming years, it is likely that generics and biosimilars will rapidly introduce new oncology drug pricing competition bringing the promise of advanced therapies to the emerging economies of SSA.

## Supporting information

S1 File Inclusivity in global research questionnaire. (DOCX)

## References

[1] Sung H, Ferlay J, Siegel RL, Laversanne M, Soerjomataram I, Jemal A, et al. Global Cancer Statistics 2020: GLOBOCAN Estimates of Incidence and Mortality Worldwide for 36 Cancers in 185 Countries. CA Cancer J Clin; 2021;71:209–49. Available from: https://pubmed.ncbi.nlm.nih.gov/33538338/ 33538338 10.3322/caac.21660

[2] de Martel C, Georges D, Bray F, Ferlay J, Clifford GM. Global burden of cancer attributable to infections in 2018: a worldwide incidence analysis. Lancet Glob Health; 2020;8:e180–90. Available from: https://pubmed.ncbi.nlm.nih.gov/31862245/ 31862245 10.1016/S2214-109X(19)30488-7

[3] Joko-Fru WY, Jedy-Agba E, Korir A, Ogunbiyi O, Dzamalala CP, Chokunonga E, et al. The evolving epidemic of breast cancer in sub-Saharan Africa: Results from the African Cancer Registry Network. Int J Cancer. Wiley-Liss Inc.; 2020;147:2131–41. doi: 10.1002/ijc.33014 32306390

[4] Parkin DM, Jemal A, Bray F, Korir AR, Kamaté B, Singh E, et al., editors. Cancer in Sub-Saharan Africa. International Agency for Research on Cancer; 2019.

[5] Sinha S, Bhatia R, Narasimamurthy M, Rayne S, Grover S. Epidemiology of Breast Cancer Presentation in Botswana, South Africa, and the United States. Journal of Surgical Research. Academic Press Inc.; 2022;279:533–9. doi: 10.1016/j.jss.2022.04.071 35868037 PMC10033457

[6] Azubuike SO, Muirhead C, Hayes L, McNally R. Rising global burden of breast cancer: the case of sub-Saharan Africa (with emphasis on Nigeria) and implications for regional development: a review. World J Surg Oncol. 2018;16:63. doi: 10.1186/s12957-018-1345-2 29566711 PMC5863808

[7] Barta JA, Powell CA, Wisnivesky JP. Global epidemiology of lung cancer. Ann Glob Health. Ubiquity Press; 2019. 10.5334/aogh.2419 PMC6724220 30741509

[8] Jedy-Agba E, Joko WY, Liu B, Buziba NG, Borok M, Korir A, et al. Trends in cervical cancer incidence in sub-Saharan Africa. Br J Cancer. Springer Nature; 2020;123:148–54. doi: 10.1038/s41416-020-0831-9 32336751 PMC7341858

[9] Seraphin TP, Joko-Fru WY, Kamaté B, Chokunonga E, Wabinga H, Somdyala NIM, et al. Rising prostate cancer incidence in Sub-Saharan Africa: A trend analysis of data from the african cancer registry network. Cancer Epidemiology Biomarkers and Prevention. American Association for Cancer Research Inc.; 2021;30:158–65. doi: 10.1158/1055-9965.EPI-20-1005 33033143

[10] Seraphin TP, Joko-Fru WY, Manraj SS, Chokunonga E, Somdyala NIM, Korir A, et al. Prostate cancer survival in sub-Saharan Africa by age, stage at diagnosis, and human development index: a population-based registry study. Cancer Causes and Control. Springer Science and Business Media Deutschland GmbH; 2021;32:1001–19. doi: 10.1007/s10552-021-01453-x 34244896 PMC8310516

[11] Ibrahim Khalil A, Franceschi S, de Martel C, Bray F, Clifford GM. Burden of Kaposi sarcoma according to HIV status: A systematic review and global analysis. Int J Cancer. John Wiley and Sons Inc; 2022. p. 1948–57. doi: 10.1002/ijc.33951 35085400

[12] Ugai T, Sasamoto N, Lee HY, Ando M, Song M, Tamimi RM, et al. Is early-onset cancer an emerging global epidemic? Current evidence and future implications. Nature Reviews Clinical Oncology 2022 19:10. Nature Publishing Group; 2022;19:656–73. Available from: https://www.nature.com/articles/s41571-022-00672-8 36068272 10.1038/s41571-022-00672-8 PMC9509459

[13] Pace LE, Mpunga T, Hategekimana V, Dusengimana J-M, Habineza H, Bosco Bigirimana J, et al. Delays in Breast Cancer Presentation and Diagnosis at Two Rural Cancer Referral Centers in Rwanda. Oncologist. 2015;780–8. Available from: doi: 10.1634/theoncologist.2014-0493 26032138 PMC4492236

[14] Bhatia RK, Narasimhamurthy M, Martei YM, Prabhakar P, Hutson J, Chiyapo S, et al. Report of clinico-pathological features of breast cancer in HIV-infected and uninfected women in Botswana. Infect Agent Cancer. BioMed Central Ltd.; 2019. doi: 10.1186/s13027-019-0245-6 31649747 PMC6805363

[15] Edmund DM, Naaeder SB, Tettey Y, Gyasi RK. Breast cancer in Ghanaian women: What has changed? Am J Clin Pathol. 2013;140:97–102. doi: 10.1309/AJCPW7TZLS3BFFIU 23765539

[16] Brown CA, Kohler RE, John O, Motswetla G, Mmalane M, Tapela N, et al. Multilevel Factors Affecting Time to Cancer Diagnosis and Care Quality in Botswana. Oncologist. Oxford University Press (OUP); 2018;23:1453–60. doi: 10.1634/theoncologist.2017-0643 30082488 PMC6292540

[17] Anakwenze C, Bhatia R, Rate W, Bakwenabatsile L, Ngoni K. Factors Related to Advanced Stage of Cancer Presentation in Botswana. J Glob Oncol. 2018; doi: 10.1200/JGO.18.00129 30532993 PMC6818282

[18] Knapp G, Tansley G, Olasehinde O, Wuraola F. Geospatial Access Predicts Cancer Stage at Presentation and Outcomes for Patients With Breast Cancer in Southwest Nigeria: A Population-Based Study. Cancer. 2021; doi: 10.1002/cncr.33394 33370458 PMC8404086

[19] Parkin DM, Sitas F, Chirenje M, Stein L, Abratt R, Wabinga H. Part I: Cancer in Indigenous Africans—burden, distribution, and trends. Lancet Oncol. 2008;9:683–92. doi: 10.1016/S1470-2045(08)70175-X 18598933

[20] Motlhale M, Sitas F, Bradshaw D, Chen WC, Singini MG, de Villiers CB, et al. Epidemiology of Kaposi’s sarcoma in sub-Saharan Africa. Cancer Epidemiol. Elsevier Ltd; 2022. 10.1016/j.canep.2022.102167 35504064

[21] Cesarman E, Damania B, Krown SE, Martin J, Bower M, Whitby D. Kaposi sarcoma. Nature Reviews Disease Primers 2019 5:1. Nature Publishing Group; 2019;5:1–21. Available from: https://www.nature.com/articles/s41572-019-0060-9 10.1038/s41572-019-0060-9 PMC6685213 30705286

[22] Cianfrocca M, Lee S, von Roenn J, Tulpule A, Dezube BJ, Aboulafia DM, et al. Randomized trial of paclitaxel versus pegylated liposomal doxorubicin for advanced human immunodeficiency virus-associated Kaposi sarcoma: evidence of symptom palliation from chemotherapy. Cancer. 2010;116:3969–77. doi: 10.1002/cncr.25362 20564162 PMC3157242

[23] Krown SE, Moser CB, MacPhail P, Matining RM, Godfrey C, Caruso SR, et al. Treatment of advanced AIDS-associated Kaposi sarcoma in resource-limited settings: a three-arm, open-label, randomised, non-inferiority trial. Lancet. 2020;395:1195–207. doi: 10.1016/S0140-6736(19)33222-2 32145827 PMC7236082

[24] Delyon J, Biard L, Renaud M, Resche-Rigon M, le Goff J, Dalle S, et al. PD-1 blockade with pembrolizumab in classic or endemic Kaposi’s sarcoma: a multicentre, single-arm, phase 2 study. Lancet Oncol; 2022;23:491–500. Available from: https://pubmed.ncbi.nlm.nih.gov/35279271/ 35279271 10.1016/S1470-2045(22)00097-3

[25] Bello JO. Natural history of castration-resistant prostate cancer in sub-Saharan African black men: a single-centre study of Nigerian men. Ecancermedicalscience. 2018;12. 10.3332/ecancer.2018.797 PMC5804715 29434663

[26] Cassell A, Yunusa B, Jalloh M, Mbodji MM, Diallo A, Ndoye M, et al. A Review of Localized Prostate Cancer: An African Perspective. World J Oncol. Elmer Press; 2019;10:162. Available from: /pmc/articles/PMC6785274/ doi: 10.14740/wjon1221 31636789 PMC6785274

[27] Cassell A, Yunusa B, Jalloh M, Ndoye M, Mbodji MM, Diallo A, et al. Management of Advanced and Metastatic Prostate Cancer: A Need for a Sub-Saharan Guideline. J Oncol. Hindawi Limited; 2019;2019. doi: 10.1155/2019/1785428 31885569 PMC6915139

[28] Fizazi K, Tran N, Fein L, Matsubara N, Rodriguez-Antolin A, Alekseev BY, et al. Abiraterone plus Prednisone in Metastatic, Castration-Sensitive Prostate Cancer. N Engl J Med. 2017;377:352–60. doi: 10.1056/NEJMoa1704174 28578607

[29] James ND, Clarke NW, Cook A, Ali A, Hoyle AP, Attard G, et al. Abiraterone acetate plus prednisolone for metastatic patients starting hormone therapy: 5-year follow-up results from the STAMPEDE randomised trial (NCT00268476). Int J Cancer; 2022; 151:422–34. Available from: https://pubmed.ncbi.nlm.nih.gov/35411939/ 35411939 10.1002/ijc.34018 PMC9321995

[30] Rush HL, Murphy L, Morgans AK, Clarke NW, Cook AD, Attard G, et al. Quality of Life in Men With Prostate Cancer Randomly Allocated to Receive Docetaxel or Abiraterone in the STAMPEDE Trial. J Clin Oncol. 2022;40:825–36. doi: 10.1200/JCO.21.00728 34757812 PMC7612717

[31] Hussain M, Mateo J, Fizazi K, Saad F, Shore N, Sandhu S, et al. Survival with Olaparib in Metastatic Castration-Resistant Prostate Cancer. N Engl J Med. 2020;383:2345–57. doi: 10.1056/NEJMoa2022485 32955174

[32] Szmulewitz RZ, Karrison T, Stadler WM, Ratain MJ. Low-Dose Abiraterone With Food: Rebutting an Editorial. J Clin Oncol. 2018;36:3060–1. doi: 10.1200/JCO.2018.79.3018 30188785

[33] Vanderpuye VDNK, Olopade OI, Huo D. Pilot Survey of Breast Cancer Management in Sub-Saharan Africa. J Glob Oncol. American Society of Clinical Oncology; 2017; 3:194. Available from: /pmc/articles/PMC5493219/ doi: 10.1200/JGO.2016.004945 28717760 PMC5493219

[34] Vanderpuye V, Grover S, Hammad N, Prabhakar P, Simonds H, Olopade F, et al. An update on the management of breast cancer in Africa. Infect Agent Cancer. BioMed Central Ltd.; 2017;12:1–12. Available from: https://infectagentscancer.biomedcentral.com/articles/10.1186/s13027-017-0124-y 28228841 10.1186/s13027-017-0124-y PMC5307840

[35] Kerr DJ, Midgley R. Can we treat cancer for a dollar a day? Guidelines for low-income countries. N Engl J Med. N Engl J Med; 2010;363:801–3. Available from: https://pubmed.ncbi.nlm.nih.gov/20818843/ 20818843 10.1056/NEJMp1002812

[36] Popli P, Gutterman EM, Omene C, Ganesan S, Mills D, Marlink R. Receptor-Defined Breast Cancer in Five East African Countries and Its Implications for Treatment: Systematic Review and Meta-Analysis. JCO Glob Oncol; 2021;7:289–301. Available from: https://pubmed.ncbi.nlm.nih.gov/33591798/ 33591798 10.1200/GO.20.00398 PMC8081496

[37] Sayed S, Moloo Z, Wasike R, Bird P, Oigara R, Govender D, et al. Is breast cancer from Sub Saharan Africa truly receptor poor? Prevalence of ER/PR/HER2 in breast cancer from Kenya. Breast. 2014;23:591–6. doi: 10.1016/j.breast.2014.06.006 25012047

[38] Mohammed SI, Harford JB. Sorting Reality from What We Think We Know About Breast Cancer in Africa. PLoS Med. Public Library of Science; 2014; 11:e1001721. Available from: https://journals.plos.org/plosmedicine/article?id=10.1371/journal.pmed.1001721 25203049 10.1371/journal.pmed.1001721 PMC4159115

[39] Joko-Fru YW, Haemmerl L, Griesel M, Mezger N, Seraphin T, Feuchtner J, et al. Breast Cancer Treatment in Sub-Saharan Africa: A Population-Based Registry Study. J Glob Oncol. American Society of Clinical Oncology (ASCO); 2018;4:20s–20s.

[40] Piccart-Gebhart MJ, Procter M, Leyland-Jones B, Goldhirsch A, Untch M, Smith I, et al. Trastuzumab after adjuvant chemotherapy in HER2-positive breast cancer. N Engl J Med. 2005;353:1659–72. doi: 10.1056/NEJMoa052306 16236737

[41] Cameron D, Piccart-Gebhart MJ, Gelber RD, Procter M, Goldhirsch A, de Azambuja E, et al. 11 years’ follow-up of trastuzumab after adjuvant chemotherapy in HER2-positive early breast cancer: final analysis of the HERceptin Adjuvant (HERA) trial. Lancet. 2017;389:1195–205. doi: 10.1016/S0140-6736(16)32616-2 28215665 PMC5465633

[42] Oriaku N, Orji M-G, Agbi R, Gimba S, Banwo-Fatai K, Nwosu T, et al. The Burden of HER-2 Positive Patients in Sub-Saharan Africa: A Caregiver Perspective. American Society of Clinical Oncology; 2018;4:155s–155s.

[43] Gershon N, Berchenko Y, Hall PS, Goldstein DA. Cost effectiveness and affordability of trastuzumab in sub-Saharan Africa for early stage HER2-positive breast cancer. Cost Effectiveness and Resource Allocation. BioMed Central Ltd.; 2019;17:1–10. Available from: https://resource-allocation.biomedcentral.com/articles/10.1186/s12962-019-0174-7 30867655 10.1186/s12962-019-0174-7 PMC6396469

[44] Galukande M, Wabinga H, Mirembe F, Karamagi C, Asea A. Molecular breast cancer subtypes prevalence in an indigenous Sub Saharan African population. Pan Afr Med J. 2014;17:249. doi: 10.11604/pamj.2014.17.249.330 25309649 PMC4189896

[45] Ssentongo P, Lewcun JA, Candela X, Ssentongo AE, Kwon EG, Ba DM, et al. Regional, racial, gender, and tumor biology disparities in breast cancer survival rates in Africa: A systematic review and meta-analysis. PLoS One; 2019;14. Available from: https://pubmed.ncbi.nlm.nih.gov/31751359/ 31751359 10.1371/journal.pone.0225039 PMC6872165

[46] Cortes J, Cescon DW, Rugo HS, Nowecki Z, Im S-A, Yusof MM, et al. Pembrolizumab plus chemotherapy versus placebo plus chemotherapy for previously untreated locally recurrent inoperable or metastatic triple-negative breast cancer (KEYNOTE-355): a randomised, placebo-controlled, double-blind, phase 3 clinical trial. Lancet. 2020;396:1817–28. doi: 10.1016/S0140-6736(20)32531-9 33278935

[47] Schmid P, Rugo HS, Adams S, Schneeweiss A, Barrios CH, Iwata H, et al. Atezolizumab plus nab-paclitaxel as first-line treatment for unresectable, locally advanced or metastatic triple-negative breast cancer (IMpassion130): updated efficacy results from a randomised, double-blind, placebo-controlled, phase 3 trial. Lancet Oncol. 2020;21:44–59. doi: 10.1016/S1470-2045(19)30689-8 31786121

[48] Cristofanilli M, Turner NC, Bondarenko I, Ro J, Im S-A, Masuda N, et al. Fulvestrant plus palbociclib versus fulvestrant plus placebo for treatment of hormone-receptor-positive, HER2-negative metastatic breast cancer that progressed on previous endocrine therapy (PALOMA-3): final analysis of the multicentre, double-blind, phase 3 randomised controlled trial. Lancet Oncol. 2016;17:425–39. doi: 10.1016/S1470-2045(15)00613-0 26947331

[49] Johnston S, Martin M, di Leo A, Im S-A, Awada A, Forrester T, et al. MONARCH 3 final PFS: a randomized study of abemaciclib as initial therapy for advanced breast cancer. NPJ Breast Cancer. 2019;5:5. doi: 10.1038/s41523-018-0097-z 30675515 PMC6336880

[50] Hortobagyi GN, Stemmer SM, Burris HA, Yap YS, Sonke GS, Paluch-Shimon S, et al. Updated results from MONALEESA-2, a phase III trial of first-line ribociclib plus letrozole versus placebo plus letrozole in hormone receptor-positive, HER2-negative advanced breast cancer. Ann Oncol. 2018;29:1541–7. doi: 10.1093/annonc/mdy155 29718092

[51] Black E, Richmond R. Prevention of Cervical Cancer in Sub-Saharan Africa: The Advantages and Challenges of HPV Vaccination. Vaccines (Basel). 2018;6. doi: 10.3390/vaccines6030061 30205561 PMC6161067

[52] Anaman-Torgbor J, Angmorterh SK, Dordunoo D, Ofori EK. Cervical cancer screening behaviours and challenges: a sub-Saharan Africa perspective. Pan Afr Med J. 2020;36:97. doi: 10.11604/pamj.2020.36.97.19071 32774656 PMC7392861

[53] Abu-Rustum NR, Yashar CM, Bean S, Bradley K, Campos SM, Chon HS, et al. NCCN Guidelines Insights: Cervical Cancer, Version 1.2020. J Natl Compr Canc Netw. 2020;18:660–6. doi: 10.6004/jnccn.2020.0027 32502976

[54] Burt LM, McCormak M, Lecuru F, Kanyike DM, Bvochora-Nsingo M, Ndlovu N, et al. Cervix Cancer in Sub-Saharan Africa: An Assessment of Cervical Cancer Management. JCO Glob Oncol. 2021;7:173–82. doi: 10.1200/GO.20.00079 33529076 PMC8081497

[55] Ndlovu N. Radiotherapy treatment in cancer control and its important role in Africa. Ecancermedicalscience. ecancer Global Foundation; 2019;13. doi: 10.3332/ecancer.2019.942 31552115 PMC6722105

[56] Reichenvater H, Matias LDS. Is Africa a ‘Graveyard’ for Linear Accelerators? Clin Oncol (R Coll Radiol). 2016;28:e179–83. doi: 10.1016/j.clon.2016.08.010 27601157

[57] Griesel M, Seraphin TP, Mezger NCS, Hämmerl L, Feuchtner J, Joko-Fru WY, et al. Cervical Cancer in Sub-Saharan Africa: A Multinational Population-Based Cohort Study of Care and Guideline Adherence. Oncologist. 2021;26:e807–16. doi: 10.1002/onco.13718 33565668 PMC8100544

[58] Colombo N, Dubot C, Lorusso D, Caceres MV, Hasegawa K, Shapira-Frommer R, et al. Pembrolizumab for Persistent, Recurrent, or Metastatic Cervical Cancer. New England Journal of Medicine. 2021;385:1856–67. doi: 10.1056/NEJMoa2112435 34534429

[59] Alexander M, Kim SY, Cheng H. Update 2020: Management of Non-Small Cell Lung Cancer. Lung. 2020;198:897–907. doi: 10.1007/s00408-020-00407-5 33175991 PMC7656891

[60] Chen R, Manochakian R, James L, Azzouqa AG, Shi H, Zhang Y, et al. Emerging therapeutic agents for advanced non-small cell lung cancer. Journal of Hematology & Oncology 2020 13:1 BioMed Central; 2020;13:1–23. Available from: https://jhoonline.biomedcentral.com/articles/10.1186/s13045-020-00881-7 32448366 10.1186/s13045-020-00881-7 PMC7245927

[61] Ohe Y, Ohashi Y, Kubota K, Tamura T, Nakagawa K, Negoro S, et al. Randomized phase III study of cisplatin plus irinotecan versus carboplatin plus paclitaxel, cisplatin plus gemcitabine, and cisplatin plus vinorelbine for advanced non-small-cell lung cancer: Four-Arm Cooperative Study in Japan. Ann Oncol. Ann Oncol; 2007; 18:317–23. Available from: https://pubmed.ncbi.nlm.nih.gov/17079694/ 17079694 10.1093/annonc/mdl377

[62] Fossella F, Pereira JR, von Pawel J, Pluzanska A, Gorbounova V, Kaukel E, et al. Randomized, multinational, phase III study of docetaxel plus platinum combinations versus vinorelbine plus cisplatin for advanced non-small-cell lung cancer: the TAX 326 study group. J Clin Oncol; 2003;21:3016–24. Available from: https://pubmed.ncbi.nlm.nih.gov/12837811/ 12837811 10.1200/JCO.2003.12.046

[63] Smit EF, van Meerbeeck JPAM, Lianes P, Debruyne C, Legrand C, Schramel F, et al. Three-arm randomized study of two cisplatin-based regimens and paclitaxel plus gemcitabine in advanced non-small-cell lung cancer: a phase III trial of the European Organization for Research and Treatment of Cancer Lung Cancer Group—EORTC 08975. J Clin Oncol; 2003; 21:3909–17. Available from: https://pubmed.ncbi.nlm.nih.gov/14581415/ 14581415 10.1200/JCO.2003.03.195

[64] Scagliotti GV, Parikh P, von Pawel J, Biesma B, Vansteenkiste J, Manegold C, et al. Phase III study comparing cisplatin plus gemcitabine with cisplatin plus pemetrexed in chemotherapy-naive patients with advanced-stage non-small-cell lung cancer. J Clin Oncol; 2008; 26:3543–51. Available from: https://pubmed.ncbi.nlm.nih.gov/18506025/ 18506025 10.1200/JCO.2007.15.0375

[65] Zatloukal P, Petruzelka L, Zemanova M, Havel L, Janku F, Judas L, et al. Concurrent versus sequential chemoradiotherapy with cisplatin and vinorelbine in locally advanced non-small cell lung cancer: a randomized study. Lung Cancer. 2004;46:87–98. doi: 10.1016/j.lungcan.2004.03.004 15364136

[66] Torre LA, Siegel RL, Jemal A. Lung Cancer Statistics. Adv Exp Med Biol. Adv Exp Med Biol; 2016;893:1–19. Available from: https://pubmed.ncbi.nlm.nih.gov/26667336/ 26667336 10.1007/978-3-319-24223-1_1

[67] Antonia SJ, Borghaei H, Ramalingam SS, Horn L, de Castro Carpeño J, Pluzanski A, et al. Four-year survival with nivolumab in patients with previously treated advanced non-small-cell lung cancer: a pooled analysis. Lancet Oncol; 2019;20:1395–408. Available from: https://pubmed.ncbi.nlm.nih.gov/31422028/ 31422028 10.1016/S1470-2045(19)30407-3 PMC7193685

[68] Garon EB, Hellmann MD, Rizvi NA, Carcereny E, Leighl NB, Ahn MJ, et al. Five-Year Overall Survival for Patients With Advanced Non‒Small-Cell Lung Cancer Treated With Pembrolizumab: Results From the Phase I KEYNOTE-001 Study. J Clin Oncol; 2019;37:2518–27. Available from: https://pubmed.ncbi.nlm.nih.gov/31154919/31154919 10.1200/JCO.19.00934 PMC6768611

[69] Singhi EK, Horn L, Sequist L v., Heymach J, Langer CJ. Advanced Non-Small Cell Lung Cancer: Sequencing Agents in the EGFR-Mutated/ALK-Rearranged Populations. Am Soc Clin Oncol Educ Book. Am Soc Clin Oncol Educ Book; 2019;39:e187–97. Available from: https://pubmed.ncbi.nlm.nih.gov/31099642/ 31099642 10.1200/EDBK_237821

[70] Lin JJ, Cardarella S, Lydon CA, Dahlberg SE, Jackman DM, Jänne PA, et al. Five-Year Survival in EGFR-Mutant Metastatic Lung Adenocarcinoma Treated with EGFR-TKIs. J Thorac Oncol. 2016;11:556–65. doi: 10.1016/j.jtho.2015.12.103 26724471 PMC4979601

[71] Pacheco JM, Gao D, Smith D, Purcell T, Hancock M, Bunn P, et al. Natural History and Factors Associated with Overall Survival in Stage IV ALK-Rearranged Non-Small Cell Lung Cancer. J Thorac Oncol. J Thorac Oncol; 2019;14:691–700. Available from: https://pubmed.ncbi.nlm.nih.gov/30599201/ 30599201 10.1016/j.jtho.2018.12.014 PMC7310567

[72] Zhao D, Chen X, Qin N, Su D, Zhou L, Zhang Q, et al. The prognostic role of EGFR-TKIs for patients with advanced non-small cell lung cancer. Scientific Reports 2017 7:1. Nature Publishing Group; 2017;7:1–9. Available from: https://www.nature.com/articles/srep40374 28079142 10.1038/srep40374 PMC5227705

[73] Ramalingam SS, Vansteenkiste J, Planchard D, Cho BC, Gray JE, Ohe Y, et al. Overall Survival with Osimertinib in Untreated, EGFR-Mutated Advanced NSCLC. N Engl J Med; 2020; 382:41–50. Available from: https://pubmed.ncbi.nlm.nih.gov/31751012/ 31751012 10.1056/NEJMoa1913662

[74] Reck M, Rodríguez-Abreu D, Robinson AG, Hui R, Csoszi T, Fülöp A, et al. Updated Analysis of KEYNOTE-024: Pembrolizumab Versus Platinum-Based Chemotherapy for Advanced Non-Small-Cell Lung Cancer With PD-L1 Tumor Proportion Score of 50% or Greater. J Clin Oncol. J Clin Oncol; 2019;37:537–46. Available from: https://pubmed.ncbi.nlm.nih.gov/30620668/ 30620668 10.1200/JCO.18.00149

[75] Gadgeel S, Rodríguez-Abreu D, Speranza G, Esteban E, Felip E, Dómine M, et al. Updated Analysis From KEYNOTE-189: Pembrolizumab or Placebo Plus Pemetrexed and Platinum for Previously Untreated Metastatic Nonsquamous Non-Small-Cell Lung Cancer. J Clin Oncol; 2020;38:1505–17. Available from: https://pubmed.ncbi.nlm.nih.gov/32150489/ 32150489 10.1200/JCO.19.03136

[76] van Eeden R, Tunmer M, Geldenhuys A, Nayler S, Rapoport BL. Lung Cancer in South Africa. J Thorac Oncol. 2020;15:22–8. doi: 10.1016/j.jtho.2019.06.032 31864550

[77] Le DT, Durham JN, Smith KN, Wang H, Bartlett BR, Aulakh LK, et al. Mismatch repair deficiency predicts response of solid tumors to PD-1 blockade. Science. 2017;357:409–13. doi: 10.1126/science.aan6733 28596308 PMC5576142

[78] Bonneville R, Krook MA, Kautto EA, Miya J, Wing MR, Chen H-Z, et al. Landscape of Microsatellite Instability Across 39 Cancer Types. JCO Precis Oncol. 2017;2017. 10.1200/PO.17.00073 PMC5972025 29850653

[79] Raskin L, Dakubo JCB, Palaski N, Greenson JK, Gruber SB. Distinct molecular features of colorectal cancer in Ghana. Cancer Epidemiol. 2013;37:556–61. doi: 10.1016/j.canep.2013.07.007 23962701 PMC4267222

[80] Irabor DO, Oluwasola OA, Ogunbiyi OJ, Ogun OG, Okolo CA, Melas M, et al. Microsatellite Instability Is Common in Colorectal Cancer in Native Nigerians. Anticancer Res. 2017;37:2649–54. doi: 10.21873/anticanres.11612 28476840

[81] Naidoo R, Tarin M, Chetty R. A comparative microsatellite analysis of colorectal cancer in patients <35 years and >50 years of age. Am J Gastroenterol. 2000;95:3266–75. 11095352 10.1111/j.1572-0241.2000.03208.x

[82] Lemery S, Keegan P, Pazdur R. First FDA Approval Agnostic of Cancer Site—When a Biomarker Defines the Indication. N Engl J Med. 2017;377:1409–12. doi: 10.1056/NEJMp1709968 29020592

[83] Marabelle A, Le DT, Ascierto PA, di Giacomo AM, de Jesus-Acosta A, Delord J-P, et al. Efficacy of Pembrolizumab in Patients With Noncolorectal High Microsatellite Instability/Mismatch Repair-Deficient Cancer: Results From the Phase II KEYNOTE-158 Study. J Clin Oncol. 2020;38:1–10. doi: 10.1200/JCO.19.02105 31682550 PMC8184060

[84] Marcus L, Lemery SJ, Keegan P, Pazdur R. FDA Approval Summary: Pembrolizumab for the Treatment of Microsatellite Instability-High Solid Tumors. Clin Cancer Res. 2019;25:3753–8. doi: 10.1158/1078-0432.CCR-18-4070 30787022

[85] Fundytus A, Sengar M, Lombe D, Hopman W, Jalink M, Gyawali B, et al. Access to cancer medicines deemed essential by oncologists in 82 countries: an international, cross-sectional survey. Lancet Oncol. 2021;22:1367–77. doi: 10.1016/S1470-2045(21)00463-0 34560006 PMC8476341

[86] Samtal C, el Jaddaoui I, Hamdi S, Bouguenouch L, Ouldim K, Nejjari C, et al. Review of prostate cancer genomic studies in Africa. Front Genet. 2022;13:911101. doi: 10.3389/fgene.2022.911101 36303548 PMC9593051

[87] Rotimi SO, Rotimi OA, Salhia B. Authorship Patterns in Cancer Genomics Publications Across Africa. JCO Glob Oncol. 2021;7:747–55. doi: 10.1200/GO.20.00552 34033494 PMC8457814

[88] Rotimi SO, Rotimi OA, Salhia B. A Review of Cancer Genetics and Genomics Studies in Africa. Front Oncol. 2020;10:606400. doi: 10.3389/fonc.2020.606400 33659210 PMC7917259

[89] Munung NS, Ambele MA, Moela P. Advancing global equity in cancer genomics—challenges and opportunities in Sub-Saharan Africa. Curr Opin Genet Dev. 2021;66:20–4. doi: 10.1016/j.gde.2020.11.006 33373832

[90] Allied Against Cancer. https://acs-prod-website.us-south.cf.appdomain.cloud/

[91] Strother RM, Gopal S, Wirth M, Chadburn A, Noy A, Cesarman E, et al. Challenges of HIV Lymphoma Clinical Trials in Africa: Lessons From the AIDS Malignancy Consortium 068 Study. JCO Glob Oncol. 2020;6:1034–40. doi: 10.1200/GO.20.00152 32634068 PMC7392773

